# Effectiveness of a Patient-Family Carer Partnership Intervention on Blood Pressure Control for People with Hypertension in Rural Communities: A Randomised Controlled Trial

**DOI:** 10.1155/2024/7033013

**Published:** 2024-05-24

**Authors:** Dejian Zeng, Wai Tong Chien, Mingyan Yang

**Affiliations:** ^1^Southern University of Science and Technology Hospital, No. 6019 Liuxian Street, Xili Avenue, Nanshan District, Shenzhen, China; ^2^The Nethersole School of Nursing, Faculty of Medicine, The Chinese University of Hong Kong, 7/F, Esther Lee Building, Shatin, Hong Kong, China

## Abstract

**Objectives:**

To examine the effectiveness of a patient-family (carer) partnership intervention on the BP control, self-care and self-efficacy for hypertensive people, and dyadic-relationship quality, depressive and anxiety symptoms, and health-related quality of life for the family dyads (hypertensive people and family carers) in rural communities of mainland China.

**Design:**

A randomised controlled trial.

**Methods:**

A total of 110 family dyads were randomly recruited from village clinics and randomly allocated to the intervention group (*n* = 55) or control group (*n* = 55). Family dyads in the control group received usual care. In addition to the usual care, family dyads in the intervention group received the individual-based, five-session patient-family (carer) partnership intervention. The primary outcomes included SBP, DBP, and the proportion of people with normal controlled BP. EuroQol five-dimensional-five-level (EQ-5D-5L) was adopted to evaluate participants' health-related quality of life. Data were collected at the baseline (T0), one-month (T1), and three-month postintervention (T2). Generalised estimating equation model was adopted to test the study hypotheses on all study outcomes.

**Results:**

Compared with the control group, hypertensive people in the intervention group had a greater reduction in SBP by 10.10 mmHg and DBP by 4.66 mmHg and a larger proportion of people with normal BP at T2, as well as statistically significant improvements at T1 and T2 in dyadic relationship, self-care, antihypertensive drug-titration rate, anxiety symptoms, and health-related quality of life. The intervention also had statistically significant positive effects on family carer's dyadic relationship and health-related quality of life at T1 and T2.

**Conclusion:**

The patient-family (carer) partnership intervention has the potential to improve hypertensive people's BP control and family dyad's dyadic-relationship quality and mental health at short-to-medium term follow-ups. *Implications for the Profession and/or Patient Care*. This study provided evidence and direction to support healthcare providers in developing and implementing patient-family (carer) partnership intervention for hypertension care in rural areas. This trial is registered with ChiCTR1900027087.

## 1. Background

Cardio-cerebrovascular disease is the leading cause of death and disability-adjusted life-year worldwide [1]. Hypertension is a major risk factor for cardio-cerebrovascular disease, and it is a major challenge for chronic illness management, with high prevalence and poorly controlled blood pressure (BP) [2]. Hypertension prevalence in rural and urban areas could be similar, but the control rates were statistically significant lower in rural areas in China and worldwide, that was, about 9.8% versus 14.5% worldwide [3] and 9.5% versus 14.0% in China [4].

The health disparities in rural areas can contribute to the lower treatment and control rates of hypertension [5]. In rural areas, the health disparities are considerably related to the healthcare system, socioeconomic condition, geographical distance of seeking healthcare services, strength and competence of healthcare providers, and individual characteristics such as education level and physical activity pattern, as well as the differences in cultural practices and living habits [6]. One of the largest health disparities presented in China is the inequality of resources and accessibility to healthcare services between rural and urban areas [7]. The percentages of hypertensive people receiving treatment provided by physicians in the recent two weeks are 11.29% in urban areas and 6.77% in rural areas [8].

Hypertension management interventions for populations in rural areas should consider specific sets of important sociodemographic characteristics of hypertensive people, the healthcare service/system, and their living districts/areas as needed. Families serving as an important source of social support for people with hypertension in rural areas can facilitate their compliance with the recommended treatment regimen [9]. Notably, Chinese family culture and values place high emphasis on the responsibilities and obligations of family members in caring for sick family members, considering them to be moral obligations and norms of Chinese societies [10]. These cultural specificities can influence family members' and carers' responses to the needs of their patients, the self-efficacy of an ill relative, and the relationship of the family dyad in daily care [11, 12].

Establishing a positive family-dyad (patient and the family carer) partnership is essential in family-oriented care at home [13]. Family care intrinsically involves the patients and their family carers (i.e., the family dyad) in close interactions and relationships. Family-dyad partnership is the supportive and collaborative relationship in illness management. A review of systematic reviews demonstrated that involving family members in chronic illness care can benefit patients and families [14]. However, there were research results showed that family care can be stressful for the patients and family carers during the caregiving process because of criticisms [15], family conflicts [16, 17], and overcontrolling or protective behaviours [18, 19], which can negatively affect the relationship between family dyad, as well as their physical and psychological health. Most family carers especially those living in rural areas often have lower levels of education and health literacy and may not be equipped or prepared for engagement and facilitated hypertension management. Interventions directed at improving family carers' ability and confidence and family-dyad partnership in providing care for hypertensive people are needed [20, 21]. Therefore, this study aimed to test a family-dyad partnership programme on BP control for people with hypertension in rural communities of mainland China.

### 1.1. Intervention Development

The development of the patient-family (carer) partnership intervention (PFPI) for people with hypertension and their family carers living in the rural areas of mainland China was (1) based on the findings of a systematic review and (2) guided by a theoretical framework-shared care model (SCM).

We conducted a systematic review to evaluate the effects of different approaches to psychoeducational intervention for family dyads in hypertension care and to examine the optimal structure, format, and components of effective interventions [22]. Synthesised evidence of the 15 (RCTs and quasiexperimental) studies demonstrated a small to medium effect of family dyad-oriented psychoeducational intervention on BP control. Several main intervention components among the effective psychoeducational interventions were identified, including education for lifestyle modification and medication adherence, home BP monitoring, and group education for family carers. In addition, interventions using a mixed-teaching approach (a combination of didactic and participatory learning) can be more effective for these family dyads than other methods. Also, several research gaps have been identified. First, very few interventions were developed to improve patient-family (carer) communication and cooperation in daily hypertension care. In addition, only two of the 15 studies adopted a theoretical framework to guide the intervention. Second, none of the included studies reported the effects of psychoeducational interventions for family dyads/family carers focused on health outcomes of family carers. Third, RCTs with high-quality methodology on psychosocial intervention for hypertension care are lacking. Well-designed RCTs of family-oriented psychoeducational intervention with commonly accepted health outcomes for the Chinese hypertension population are recommended. Finally, only two studies on hypertension care were conducted in rural areas; thus, the effects of psychoeducational intervention for hypertensive patients residing in rural areas are uncertain and inconclusive. Considering the low percentage of well-controlled hypertension and health disparities (e.g., lack of healthcare providers) in rural communities such as remote districts or villages in mainland China, there is a strong need or demand for a well-prepared family carer to facilitate and support daily care, lifestyle change, and health management for their hypertensive relatives. Therefore, a family-dyad psychoeducational intervention with didactic and participatory learning strategies (e.g., family dyadic communication, decision-making, and action planning) for hypertension care in rural communities can be designed for further testing among the population of family dyads of people with hypertension.

**Figure 1:**
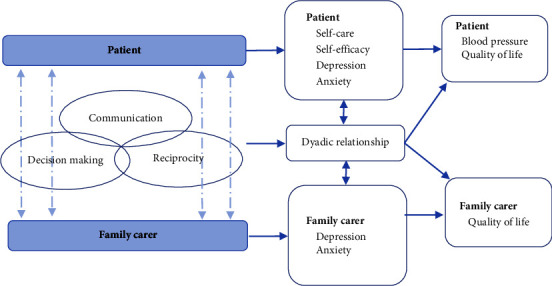
Theoretical framework of this study.

SCM was adopted to guide the intervention used in this study. SCM provides a structure on how to involve a family carer in caring for a patient with chronic illness.  Figure 1 presents the theoretical framework of the current study. In the SCM, shared care is defined as an interpersonal process used by patients and family carers (family dyad) in home care to exchange support and manage a chronic illness [23]. Shared care is a dyadic process in which each participant affects and is affected by the other(s) [24]. The SCM hypothesises and anticipates that shared care can improve the effects of providing and receiving a family member's (carer's) assistance on improving the quality of the dyad's relationship and therefore positively affects the self-care and promotes the physical and mental health of the family dyads [23, 25]. Shared care elements (communication, decision-making, and reciprocity) have substantial positive associations with the self-care, depressive symptoms, dyadic-relationship quality, and health-related quality of life of patients with heart diseases and their family carers [26–28]. The elements or components of shared care also have inverse relationships with the strain and depressive symptoms of patients and their family carers (family dyads) [25]. Therefore, the SCM provided a practical and structured framework for developing the patient-family carer partnership intervention for the current study. The three main elements of shared care (communication, decision-making, and reciprocity) were used to guide intervention development.

## 2. Method

### 2.1. Study Design

This study adopted a single-blinded RCT with a parallel control (usual care) group and repeated measurements at one- and three-month postintervention. The study is reported in line with the Consolidated Standards of Reporting Trials 2010 Statement. We registered the study in the Chinese Clinical Trial Registry (registration number: ChiCTR1900027087) in October of 2019.

This study hypothesised that the participants in the intervention group would show statistically significant greater improvements at one-month and three-month postintervention, when compared with those in the control (usual care) group, on the followings:

1. The patients' SBP and DBP levels and proportion of patients with normal controlled BP (primary outcomes), self-care, self-efficacy, antihypertensive drug-treatment rate, antihypertensive drug-titration rate, dyadic-relationship quality, depressive and anxiety symptoms, and health-related quality of life
2. Family carers' dyadic-relationship quality, depressive and anxiety symptoms, and health-related quality of life

### 2.2. Participants and Setting

Hypertensive patients receiving home visits and care at two village clinics in Liuyang City, Hunan Province, China, were the potential eligible participants of this study. The eligibility of participants was identified according to the study criteria by the researcher in home visit. People with hypertension and their family carers (dyads) were randomly enrolled in this study.

These two adjacent rural villages are located in Liuyang City, which is the most populous county-level division in the easternmost part of Hunan Province of mainland China. Meanwhile, Hunan Province is located in the south-central part of China, with over 69 million population. Liuyang city (covering an area of around 5,000 km^2^) comprises 1.5 million residents. The villages under study are Gaoping and Longquan villages in Gaoping county, which are remote villages on the east side of Liuyang, being 20–30 km distance from Liuyang City. Each village has over 2,000 residents. Owing to the distance from the city, the village residents usually receive healthcare services provided by doctors at the village clinics. The public village clinics have only one medical staff (village doctor) in each clinic who provides primary health care to all residents there, such as home visits and medical consultations for people with hypertension, diabetes, and other chronic illnesses in the village(s).

Random sampling was used to recruit the eligible participants. The researcher reviewed the hypertensive people's medical records in the two village clinics under study and created a list of potential participants in alphabetical order of their family names after screening. The people on the list were randomly selected using a random number table and approached by the researcher during home visits to confirm the study eligibility and ask for consent for participation. The block randomisation with a block size of four or six and sealed opaque envelopes labelled with group (number) were adopted to ensure the randomisation and allocation concealment, respectively. An independent research assistant prepared the randomisation schedule and sealed opaque envelopes. The randomisation sequence was generated from an online randomisation programme (https://www.sealedenvelope.com). After baseline measurement, participants opened the sealed envelope and were allocated to either the intervention or the control group, which was concealed from the outcome assessors, as well as the clinic staff. The inclusion criteria for people with hypertension were as follows:

1. Those aged 18 years or above.
2. Having essential hypertension without adequate BP control (SBP ≥140 mmHg and/or DBP ≥90 mmHg). Essential hypertension was confirmed by examining the patient's medical records in the village clinics. When “essential” was not clearly marked, the village doctor would check against the patient' medical information in the clinic for interpretation and confirmation.
3. Living with one or more family members.
4. Speaking Mandarin or local dialect.

People were excluded if they were as follows:

1. Diagnosed with a terminal illness (e.g., cancer, end-stage renal disease, and severe heart failure)
2. Diagnosed with a mental disorder, including dementia, schizophrenia, etc.
3. Diagnosed with stroke or COPD
4. Having physical disability, which was defined as needing assistance with or inability in any of the six activities of daily living (e.g., toilet, feeding, dressing, grooming, physical ambulation, or bathing) in the Physical Self-maintenance Scale [29]
5. Living alone
6. Participating or having participated recently in a structured hypertension management programme in the last six months

A family carer is a member of a family with a kinship, marital, or coresidence relationship involved in a patient's daily health care [30]. Each person nominated one family carer for participation by asking two questions in the sociodemographic datasheet: (1) “Do you have family member(s) who get involved with your health care in helping with medications, blood pressure monitoring, clinic visits, smoking cessation, alcohol control, weight loss, healthy diet, sodium restriction, or physical activity?”; and (2) “How long do you two spend together every day on average”? The family member who provided more assistance and stayed longer time was selected as the family carer to be enrolled in this study.

The family carers were included if they were as follows:

1. Aged 18 years or above
2. Blood, by-marriage, or coresidence relatives of the patient
3. Contactable by phone or WeChat

Conversely, the carers were excluded if they were as follows:

1. Having a severe mental disorder such as dementia, schizophrenia, acute or severe depression/anxiety disorders, and/or learning disorder
2. Taking care of two or more patients in the family
3. Diagnosed with hypertension

### 2.3. Sample Size

The sample size was estimated with reference to the effect sizes on the primary outcomes (change in SBP, DBP, and proportion of normal controlled BP) in similar studies. In our previous systematic review [22], the Cohen's effect size (d) of family dyad-oriented psychoeducational intervention for hypertensive people on changes in SBP and DBP was 0.59 and 0.57, respectively, at short-term (immediately to three months) postintervention. From the results, the sample size could be 94 and 100, respectively, with a two-group comparison test such as *t*-test (two-tailed) with 80% power at the statistically significant level of 5%, by using G*∗*Power [31]. Moreover, the results of our previous systematic review reported attrition rates between 0% and 19.77%, and most studies had an attrition rate below 10% (13 out of the 15 included studies) [22]. Furthermore, the pilot study of this RCT indicated an attrition rate of 4.55% [32]. Therefore, an attrition rate of 10% was used, and the final sample size was 110 family dyads (i.e., 55 for each group).

### 2.4. Intervention Group

The people with hypertension and their family carers received the patient-family (carer) partnership intervention (PFPI), which were five individual-based, biweekly face-to-face training sessions. We conducted a pilot study to test the feasibility, acceptability, and preliminary effect of the patient-family (carer) partnership intervention for people with hypertension in a Chinese rural community. The findings of this pilot study indicated that the PFPI was a feasible and acceptable programme, as reflected by its high recruitment and intervention and study-completion rates, as well as positive feedback and perceived benefits from the participants. The results of the pilot study and the structure, content, format, and components of the PFPI have been published somewhere [32]. Improvements in the intervention in current RCT were made based on the findings of pilot study.  Table 1 outlines the protocol of the PFPI.

In this study, the PFPI was delivered by the first author, who is a registered nurse and has research experience in chronic disease management (e.g., cardiovascular disease, stroke, and diabetes) in rural communities. Moreover, the researcher could speak the local language of the participants and was familiar with their local culture. This familiarity could facilitate the researcher-participant communication in the delivery of the intervention and in sample recruitment.

### 2.5. Control Group

Participants in the control and intervention groups received usual care delivered by a village doctor during home visits every three months following government regulations: the village doctor monitored BP, provided advice on hypertension self-care, and responded to people's questions about hypertension management during home visits. The BP values collected were uploaded to the hypertension follow-up system managed by the Health Bureau of Liuyang City. The village doctors did not prescribe antihypertensive drugs during home follow-up. Patients who needed prescriptions were referred to visit the village clinic or other regional clinics/hospitals for medical care.

### 2.6. Instruments

#### 2.6.1. Demographic Data Form

The demographic data form was developed and used to collect the sociodemographic and clinical characteristics of people with hypertension and their family carers. The sociodemographic and clinical information of people with hypertension included age, gender, marital status, employment status, education level, body mass index (BMI), smoking status, alcohol status, physical exercise, medical insurance, annual family income, family structure/unit, duration of hypertension, and comorbidities. The family carers' age, gender, marital status, employment status, education level, BMI, smoking status, alcohol status, medical diseases, and the relationship with patient were also collected.

#### 2.6.2. Measure for Outcomes Variables

*(1) BP Measurement*. The method and procedure for measuring BP followed the Chinese hypertension management guideline [33]. Before starting to measure BP, the patients sat quietly in a chair, feet on the floor, and back supported, for at least 5 minutes; exercise and smoking were avoided for at least 30 minutes before measurement; the bladder was emptied; and the patient's arm was supported (resting on a desk). The middle of the cuff was placed on the patient's upper arm in the same horizontal position as the right atrium which is at the midpoint of the sternum. The correct cuff size adhering to Whelton et al. [34] recommendation was used. An electronic upper-arm sphygmomanometer (OMRON HEM-752) with a validated measurement protocol and the results have been published in a peer-reviewed journal was used to measure BP [35]. BP was measured in both arms, and the arm with a higher BP reading was used for all subsequent BP measurements. BP measurement was repeated at an interval of 1 min, and the mean value of two readings was recorded. If the difference between two readings of SBP or DBP was more than 5 mmHg, it was measured again, and the average value of three readings was recorded [33].

*(2) Proportion of People with Normal BP*. The number of hypertensive people with normal BP at each assessment divided by the total number of hypertensive people enrolled in the group resulted in the proportion of people with well-controlled or normal BP at that time of measurement. According to the Chinese hypertension management guideline [33], the normal BP level of Chinese adults was below 140/90 mmHg (i.e., both SBP and DBP should achieve this norm-based standard); for patients aged 65 years or above, a normal BP should be less than 150/90 mmHg; and for patients with diabetes, it should be less than 130/80 mmHg. The determination of normal BP control was based on the current BP reading taken by the researcher/data collector in T0, T1, and T2.

*(3) Antihypertensive Drug-Treatment Rate*. The assessor asked participants a single question (e.g., “Have you taken any antihypertensive drugs prescribed by your doctor in the past two weeks?”) to collect data about the antihypertensive drug usage in the past two weeks. The antihypertensive drug-treatment rate refers to the percentage of total number of patients took antihypertensive drugs (in the past two weeks) among the hypertensive patients who participated in this study.

*(4) Antihypertensive Drug-Titration Rate*. The assessor asked participants the question “Have your antihypertensive drugs prescription been adjusted by your doctor in the past two months?” to collect data about the titration of the antihypertensive drug. The antihypertensive drug-titration rate refers to the percentage of the total number of patients who had drug titration (in the past two months) among the hypertensive patients who participated in this study.

*(5) Hypertension Self-Care Profile (HBP SCP)*. Three scales are included in HBP SCP (behaviour, motivation, and self-efficacy), which can be used together or independently. In this study, the Behaviour and Self-efficacy subscales in the HBP SCP were used to measure hypertensive people's self-care behaviour and self-efficacy, respectively. Each subscale contains 20 items rated on a four-point scale; a higher score indicates a high level of self-care behaviour or self-efficacy. The Chinese version of HBP SCP revealed good psychometric properties in the Chinese hypertensive population. Cronbach's alpha coefficients of the Behaviour and Self-efficacy subscale were 0.86 and 0.93, respectively. Moderate correlations were identified between HBP SCP subscales and Treatment Adherence Questionnaire for Hypertension (TAQPH) scales (*r* = 0.45, 0.61, 0.65, all *p* < 0.001) [36].

*(6) Dyadic Relationship Scale (DRS)*. A Chinese version of the DRS was used to measure the family dyad's partnership quality. It comprised two separate versions, namely, the Patient and the Caregiver version, to evaluate both the patients' and caregivers' perceptions towards the impacts of family care on positive and negative (dyadic strain) interactions [37]. The Patient and Caregiver versions of the DRS were rated on a four-point Likert scale, from 0 (strongly agree) to 3 (strongly disagree). A lower score of the two versions indicated better perceived dyadic relationships by patients and their family carers, respectively. The Chinese versions were tested with 132 hypertensive people and their family carers in China, with good internal consistency (Cronbach's alphas = 0.82 and 0.83 for the Patient and Caregiver version, respectively), positive correlations with the self-efficacy subscale of the Hypertension Self-Care Profile and Zarit Burden Interview schedule (Pearson's *r* = 0.70 and 0.79, respectively; both *p* < 0.001), and test-retest reliability (ICC = 0.97 and 0.96, both *p* < 0.01) (Zeng, Yang, and Chien, 2022).

*(7) Patient Health Questionnaire-9 (PHQ-9) and Generalised Anxiety Disorder Scale-7 (GAD-7)*. The PHQ-9 and GAD-7 are efficient tools for measuring perceived depressive and anxiety symptoms, respectively. Items in PHP-9 and GAD-7 are rated on a four-point Likert scale from 0 “never” to 3 “almost every day;” and the higher scores of both scales indicated more severe symptoms of depression and anxiety, respectively. Cronbach's alpha coefficient of the Chinese version of PHQ-9 was 0.82 in Chinese rural elderly [38]. The GAD-7 has been translated into a Chinese version and validated in general hospital outpatients, with a Cronbach's alpha coefficient of 0.90 and test-retest reliability of 0.86 [39].

*(8) EuroQol Five-Dimensional-Five-Level (EQ-5D-5L)*. EQ-5D-5L, including the descriptive system and the EQ visual analogue scale (EQ-VAS), was adopted to evaluate participants' HRQoL. Five dimensions of individual health condition (e.g., mobility, self-care, usual activities, pain/discomfort, and anxiety/depression) were measured by the descriptive system using five items with a five-point Likert scale from 0—“No” to 5—“Extreme”. The mainland China set value of EQ-5D-5L was used to convert the ratings on five dimensions into a single value, in which a higher score represented poorer HRQoL [40]. The EQ-VAS was a 20 cm vertical, visual analogue scale rating on one's overall health, with an endpoint between 0 (worst imaginable health state) and 100 (best imaginable health state). In Chinese patients with chronic illnesses, Cohen's kappa for the test-retest reliability of the self-classifier ranged from 0.41 to 1.00. Validity was demonstrated using known-group construct validity: seven of 10 priori hypotheses relating the EQ-5D dimensions to SF-36 dimensions were fulfilled [41].

### 2.7. Process Evaluation

A checklist of all items of the intervention protocol to check/monitor the intervention fidelity was used by the researcher during each session to monitor the intervention implementation (see supplementary file 1). The family dyads' adherence to the partnership techniques in daily hypertension care is difficult to assess. In this study, strategies, such as behaviour observation during sessions and reporting and the feedback sought from the participants (dyads), were used to assess the family dyad's adherence to the learned partnership and related techniques in daily hypertension care. For example, the researcher asked the family dyad to provide examples of the communication, decision-making, and reciprocity used in hypertension care [28] and observed the dyad' s communication styles and patterns during the training sessions. The identified partnership issues were recorded in the “checklist of partnership skills implementation during training sessions and daily life.” Each identified partnership issue was listed and marked as “implemented” or “non-implemented/inappropriately implemented” by the researcher. Further training was implemented in the next or subsequent sessions to address the “non-implemented/inappropriately implemented” skills.

### 2.8. Data Collection

The researcher reviewed a total of 702 patients' medical records in two village clinics (387 and 315 records per clinic) under study at Liuyang City, Hunan Province, China, between July and August 2020 and created a list of 530 potential participants in alphabetical order of their family names. There were 180 (33.96%) patients on the list, who were randomly selected and approached during home visits to confirm the study eligibility and ask for consent for participation. After visiting 137 patients, 110 eligible family dyads (patients and their family carers) agreed to participate in the study. The 110 dyads were randomly assigned to either the intervention or control group in equal total numbers in each group, that is, with 55 dyads per group. The recruitment was conducted by the researcher.

Written informed consent was obtained from the participants who agreed to participate in this study. Then, the researcher collected the baseline data (T0). For the participants who could not read and understand the items/contents in instruments, the researcher/assessors read and explained the items and recorded participants' responses to each item.

The researcher delivered the ten-week PFPI from August to October 2020. Postintervention data collection was conducted through home visits at 1-month (T1, November 2020) and 3-month (T2, January 2021) postintervention by a retired village doctor who was blinded to the group allocation. The retired village doctor was trained (in the village clinic) by the researcher to develop skills in data collection. Five hypertensive people were invited to participate in the training session. Peer assessments were performed on these five people with hypertension to ensure BP measurement consistency between the researcher and the assessor. An explanation of the instruments was provided during the assessor training. Considering some of the participants in the rural communities were illiterate, the accessor practiced reading and interpreting each item in the instruments to the participants. The practices for data collection were conducted in the training session until the assessor was competent to collect the data independently.

### 2.9. Data Analysis

IBM SPSS version 25.0 (IBM Corp. Armonk, NY, USA) was used for data analysis. Data cleaning was adopted to ensure data quality and study validity. All statistic tests involved were two-sided with the statistically significant level set at 0.05. Descriptive statistics were used to summarise the participants' demographic and clinical characteristics and outcome variables. The assessment of normality for the continuous variables was conducted using Q-Q plot, skewness, and kurtosis statistics.

Chi-square tests and independent *t*-tests were adopted to test the homogeneity of the sample at baseline measurement. Chi-square test or Fisher's exact test was used for categorical variables. Independent *t*-tests were applied for the continuous variables that followed a normal distribution.

Intention-to-treat (ITT) analysis was used for data or outcome analysis. ITT analysis method could preserve the sample size and statistical power, thereby resulting in a more accurate, unbiased estimation of the effectiveness of an intervention [42]. The generalised estimating equation (GEE) model was adopted to analyse the group and time differences of individual outcome variables. In the current study, the data collected at three different time points within the same subject were usually auto-correlated. Therefore, the AR(1) model was selected as working matrices in GEE analyses. The GEE model can be applied when the data missing completely at random (MCAR) [43]. In this study, the results of Little's MCAR test (*χ*^2^ = 108.949, DF = 148, *p*=0.993) supports the MCAR and GEE can be used.

In the GEE analyses, the interaction (group × time) treatment effects were included in the GEE model to examine the changes in each outcome between the intervention and control groups across the three time points. When the interaction treatment effect was statistically significant in the GEE analysis, pairwise contrast tests were used to identify any statistically significant mean score differences in each outcome measure between groups at postintervention time points (T1 and T2).

Incorporating covariates into the GEE model could minimise the bias of confounding variables and safeguard the statistical power of the intervention effects [44]. In this study, potential covariates were identified based on the results of comparisons for sociodemographic and clinical characteristics and outcome variable scores at baseline between the intervention and control groups. Variables with *p* < 0.1 were considered as potential covariates and adjusted in the GEE model [45].

### 2.10. Ethical Considerations

This study was conducted in compliance with the principles outlined in the Declaration of Helsinki. Approvals for this study were obtained from the University Cluster Clinical Research Ethics Committee (CREC Ref. No.: 2019.375). Access permission for this study from the village clinic under study was also obtained. Participant recruitment was followed the voluntary principle. Each potential participant (hypertensive people and their family carers) received a detailed introduction about the study. Informed written consent was obtained from all individual participants before collecting any data. All participants had the right to refuse to participate or withdraw from the study without any influence on usual care services provided by clinical doctors. All data collected were anonymous, kept confidential, and used for research purposes only. The participants were not in any way identifiable in the research reports.

## 3. Results

**Figure 2:**
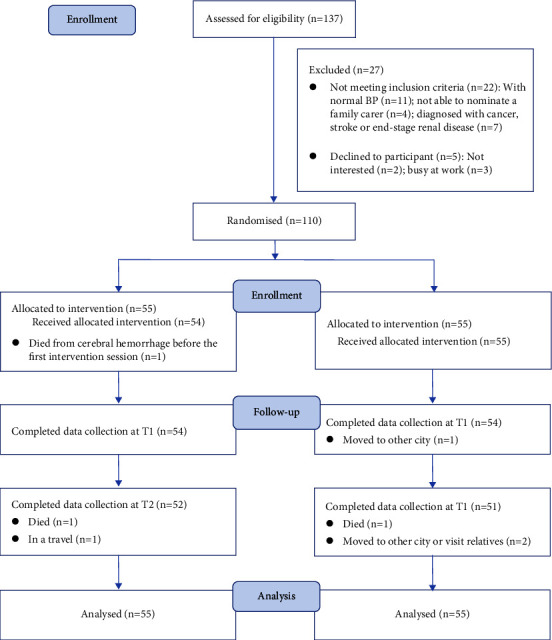
The flowchart of participant recruitment and allocation.

110 eligible family dyads (patients and their family carers) participated in the study. Within one month after the intervention (T1), one person with hypertension in the intervention group died after baseline data collection; one family dyad from the control group moved to the city to live with their son. Therefore, the two groups shared the same attrition rate of 1.82% at T1. At three months postintervention (T2), one person with hypertension died after T1 in both groups; one dyad in the intervention group and two dyads in the control group were out of villages at the time-point of T2. Therefore, the attrition rates at T2 were 5.45% (*n* = 3) for the intervention group and 7.27% (*n* = 4) for the control group; hence, the attrition rate of the study was 6.36% (*n* = 7) at T2.  Figure 2 illustrates the flowchart of participants' recruitment, group allocation, and number and reasons for withdrawals.

### 3.1. Demographic and Clinical Characteristics

Table 2 presents the demographic and clinical characteristics of people with hypertension. The mean age was 67.65 years (SD = 11.53). The majority of them were female (60.91%, *n* = 67), married (72.73%, *n* = 80), and farmers (93.36%, *n* = 106). 30.00% of the people with hypertension were illiterates (*n* = 33). The mean BMI was 23.52 kg/m^2^ (SD = 3.61). About 15% (*n* = 17) and 35% (*n* = 39) were currently drinking and smoking, respectively. More than half of the people with hypertension (54.55%, *n* = 60) took part in farm work. Their average duration of hypertension was 6.25 years (SD = 3.64). About two-thirds of people with hypertension had at least one comorbidity (60.91%, *n* = 67), from whom one-third of them were diagnosed with diabetes (29.09%, *n* = 32). No statistically significant differences were observed in the demographic and clinical characteristics of people with hypertension between the two groups (*p* values ranged from 0.06 to 1.00).

The demographic characteristics of family carers are summarised in Table 3. About two-thirds of the family carers were spouses of people with hypertension (67.27%, *n* = 74). The average age of family carers was 59.51 years (SD = 9.53), and nearly two-thirds of them (61.82%, *n* = 68) were male. The majority of them completed at least primary school education (66.36%, *n* = 73) and farmers (89.09%, *n* = 98). Their average BMI was 24.91 kg/m^2^ (SD = 2.78). About half of them (49.09%, *n* = 54) had one or more medical illnesses. Neither the Chi-square tests nor *t*-tests identified statistically significant differences in the demographic and clinical characteristics of family carers between the two groups.

### 3.2. Outcome Variables at Baseline

The mean scores or frequencies/percentages of the outcome variables are summarised in Table 4. At baseline (T0), the mean SBP values for both study groups were above the normal level of SBP (90–140 mmHg), and the mean DBP values were normal (60–90 mmHg). People with hypertension in the two groups had similar mean score of DRS-C-PT, 12.60 (SD = 3.45) for the intervention group and 12.22 (SD = 3.90) for the control group. The results of independent *t*-tests showed no statistically significant differences between groups in the outcome variables of people with hypertension, including SBP, DBP, dyadic-relationship quality, self-care, self-efficacy, depressive symptoms, anxiety symptoms, and health-related quality of life at T0, with *p* values ranging from 0.29 to 0.89; the Chi-square tests did not identify any statistically significant differences in antihypertensive drug-treatment rate (*p*=0.70) and the rate of hypertensive drugs titration (*p*=0.54) at T0.

All the outcome variables of family carers were computed using independent *t*-tests. There were no statistically significant differences between groups in family carers' dyadic-relationship quality, depressive symptoms, anxiety symptoms, and health-related quality of life at T0, with *p* values ranging from 0.44 to 0.98.

### 3.3. Effects of PFPI on People with Hypertension

The results of outcome variables for people with hypertension and family carers at T0, T1, and T2 are presented in Table 4. Most of the outcome variables were normally distributed with standard skewness and kurtosis scores between −1.96 and + 1.96 and the data points were approximately located on the diagonal line of the Q-Q plot. However, the scores of PHQ-9 and GAD-7 for patients were not normally distributed. The normality of the PHQ-9 and GAD-7 scores across the three data collection time points was met with logarithm transformation. The effect sizes for patients' continuous outcome variables at T1 were small to medium, with Cohen's *d* values ranging from 0.33 to 0.77; at T2, Cohen's *d* values ranged from 0.31 to 0.92. Regarding the categorical outcome variables of patients, the odds ratio ranged from 1.49 to 11.81 at T1 and 1.51 to 9.91 at T2. For the outcome variables of family carers, the effect sizes were small, with Cohen's *d* values ranging from 0.18 to 0.44 at T1 and 0.20 to 0.44 at T2.

The results of GEE test (Table 5) indicated statistically significant interaction (group × time) treatment effects on the study outcomes, including SBP, DBP, proportion of people with normal BP, DRS-C-PT, HBP SCP-Behaviour, HBP SCP-Self-efficacy, PHQ-9, GAD-7, EQ-5D-5L index score and EQ-VAS of people with hypertension with *p* values ranged from <0.001 to 0.04, between the two groups over three-month follow-up. Regarding the effects of PFPI on family carers, the results of the GEE test (Table 5) indicated statistically significant interaction treatment effects on GRS-C-CG, GAD-7, EQ-5D-5L index score, and EQ-VAS between groups over the three-month follow-up, with the *p* values ranged from <0.001 to 0.03.

Pairwise contrast tests in the GEE (Table 5) indicated that, compared with the control group, the people with hypertension in the intervention group reported statistically significant greater improvements in SBP, DBP, DRS-C-PT, HBP SCP-Behaviour, hypertension drug-titration rate, GAD-7, EQ-5D-5L index score, and EQ-VAS at T1 and T2; and in the proportion of people with normal BP, HBP SCP-Self-efficacy at T2. When compared with the control group, the family carers had statistically significant greater improvements in GRS-C-CG and EQ-5D-5L index score at T1 and T2, and in GAD-7 and EQ-VAS at T1.

## 4. Discussion

### 4.1. Effects on the Outcomes of People with Hypertension

We supported the study hypotheses that people with hypertension in the intervention group would show statistically significant greater improvements in SBP and DBP values, dyadic relationship, self-care, hypertensive drug-titration rate, anxiety symptoms, and HRQoL at both T1 and T2 and on the proportion of patients with normal BP and self-efficacy at T2, when compared with those in the control (usual care) group. However, the hypotheses concerned with statistically significant greater improvements on the proportion of people with normal BP and self-efficacy at T1 and on antihypertensive drug-treatment rate and depressive symptoms at both T1 and T2 were not supported.

#### 4.1.1. SBP and DBP Values and the Proportion of People with Normal BP

The effect sizes of PFPI on SBP were medium (Cohen's *d* = 0.77) at T1 and large (Cohen's *d* = 0.92) at T2, whereas those on DBP were small at T1 and T2, with Cohen's *d* = 0.44 and 0.47, respectively. The findings of BP control are consistent with and more effective (in SBP and DBP reduction) than the results of our previous systematic review in which eight included RCTs of family dyad-oriented psychoeducational interventions reported that the SBP and/or DBP of people with hypertension statistically significant decreased at less than three months postintervention (medium pooled effect sizes of 0.59 and 0.57, respectively) [22]. Furthermore, PFPI significantly improved the proportion of people with normal BP across the study period, from zero at baseline to 23.64% at T1 and 41.82% at T2. Among the studies included in our previous systematic review [22], four reported the effects of family support interventions on the proportion of people with normal BP. The improvements in the proportions of people with normal BP in these studies (15.8%–44.90%) were similar to those in the current study (23.64% at T1 and 41.82% at T2). The overall rate of well-controlled hypertension (SBP <130 mmHg and DBP <80 mmHg) in the USA in 2017–2018 was 39.64% [46]. Therefore, PFPI could be useful in improving the proportion of people with normal BP across rural areas, bringing it to the same level in developed countries.

People with hypertension in rural communities could not receive adequate healthcare services [7, 8]. In the current study, the clinic doctor (the only staff in the clinic) needs to provide nearly 300 home visits each month to all patients with hypertension and/or diabetes in the village. The large workload is not conducive to providing complete and effective hypertension care services. The family carers in the PFPI group were trained to supervise and assist in patients' medication intake, BP monitoring, and problem behaviour change and to record BP values and medication adherence in the booklet provided for reference to healthcare providers. Therefore, they were employed as surrogates for formal healthcare providers [47]. This assistance in hypertension care could alleviate the effect of the shortage of healthcare providers on hypertension management in rural areas [48]. Moreover, compared with the previous studies on family-oriented or family support intervention [48, 49], PFPI was delivered in face-to-face individual (for family dyad) sessions during home visits. The scattered living status and underdeveloped transportation in rural areas could prevent patients from actively seeking healthcare services. The healthcare service (e.g., PFPI) delivery format of home visits addresses the health disparities in seeking healthcare service for rural community residents due to long geographical distance.

The construct of SCM, which was adopted for PFPI development in this study, could interpret the statistically significant effect of PFPI in BP control. The three main components (communication, decision-making, and reciprocity with family dyad) in SCM were committed to building an effective patient-family carer (dyadic) partnership in daily hypertension care. The conflicting beliefs, difficulty in talking about an emotional topic, and lack of skills in handling conflict negatively affected the care partnership [15, 16]. PFPI provided dyadic communication skill training improving dyadic communication led to reductions in depressive symptoms for the patients and/or family carers and a positive value for the family. Systematic reviews demonstrated that family-dyadic partnership-/relationship-focused interventions were more likely to lead to reductions in depressive symptoms for patients with chronic illness and their family carers than the family interventions only directed at increasing knowledge and skills for managing the disease [47]. The exchange of values and prefers about self-management strategies between the dyad members is the basis of decision-making and action in hypertension care; it could improve the self-care (e.g., medication intake and problem behaviour change) of patients with hypertension [50]. Implementing the action plan with mutual support is highly emphasised in the SCM and PFPI [28]. In the PFPI sessions, the family dyad was guided to identify problem behaviour and set goals; discuss the influence of behaviour change; discuss difficulties in behaviour changes and effective coping strategies; identify ways to seek information, professional help, and community support whenever needed; and formulate an action plan. The family dyad applied these decision-making techniques in daily hypertension care situations when needed to make decisions. Research has demonstrated that the improved decision-making skill of stroke survivor-caregiver dyad was positively associated with survivors' depressive symptoms, dyadic relationship, and survivor coping skills [51]. Dyadic partnership is also a process of giving and accepting physical and emotional help with appreciation [52]. For example, in the PFPI sessions, the family dyads discussed the assistance provided by the family carer and received by the patient in behaviour modification. They also discussed the difficulties and challenges throughout the process of giving and receiving assistance and the strategies to overcome them and express gratitude and appreciation. These reciprocity skills facilitate the family dyad's mutual cooperation in BP monitoring, medication adherence, and behaviour change at home.

Another feature of PFPI is cultural sensitivity. Interventions taking the culture into account are more likely to lead to more positive behavioural changes [53]. In the rural areas of China, family culture and values highly emphasise the responsibilities and obligations of family members in caring for sick family members [10]. Therefore, in PFPI, the family carer was treated as a partner in hypertension care. Each family dyad worked together to promote treatment adherence and lifestyle modification through effective dyadic communication (to avoid conflicts and over-protection) and decision-making in health care.

#### 4.1.2. Dyadic-Relationship Quality

In this study, the dyadic relationship significantly improved for the intervention group at T1 (*p*=0.004) and T2 (*p* < 0.001) with medium effect sizes (Cohen's *d* = 0.52 and 0.65, respectively). As the dyadic relationship quality was seldom measured as an outcome in previous studies [47], limited information about the effect of interventions on dyadic relationship quality among people with hypertension could be found. Seber and Woda [28] developed a shared care dyadic intervention based on SCM for patients with heart failure and used a dyadic relationship measure as one of the patient outcomes in their pilot study. However, noticeable differences were found in the participants, sample size, study design, and times of outcome measurements between two studies, which may have led to the difference in their effect sizes on the dyadic relationship quality, with a Cohen's *d* of 0.65 at three-month postintervention in the current study and 0.25 immediately postintervention in Sebern and Woda's study [28]. In an RCT study, a family partnership intervention was effective in improving perceived confidence and motivation for self-care and reducing dietary sodium among heart failure patients. Other RCT study also demonstrated the effectiveness of family-focused dyadic psychoeducational intervention on the dyadic relationship of stroke survivors and their family caregivers [51]. Therefore, family-dyad partnership intervention may be feasible and effective in improving self-care and self-efficacy for patients with chronic illnesses.

#### 4.1.3. Self-Care and Self-Efficacy

PFPI significantly improved hypertensive people's self-care and self-efficacy in hypertension management with medium and small effects, respectively (Cohen's *d* = 0.65 and 0.31, respectively). In our previous systematic review [22], three studies reported the results of self-care, all of which revealed statistically significant improvements. Similar to this study, educating family dyad jointly in the educational sessions on knowledge and skills of lifestyle modifications through discussion and goal setting and encouraging family carers to supervise patients' behavioural changes were used in previous studies. These intervention components/contents were also demonstrated (in a systematic review) to be effective in improving self-management for patients with uncontrolled type II diabetes mellitus [54]. PFPI also adopted participatory learning strategies to improve the family dyads' skill in identifying the problem behaviour and making decisions and action plans for behaviour change. The participatory learning strategies encourage more active involvement of participants in the learning process, improve the dyadic communication and cooperative techniques of family dyad in health care, and facilitate changes in their lifestyle behaviour [55].

#### 4.1.4. Antihypertensive Drug-Treatment Rate and Antihypertensive Drug-Titration Rate

In the current study, the hypertensive people in the intervention and control groups had improved antihypertensive drug-treatment rates. However, the difference between groups was nonsignificant (*p*=0.17 and 0.18, respectively) at T1 and T2. In the home visit service (usual care), the village doctor monitored the BP of people with hypertension (every three months) and encouraged them to intake hypertensive drugs for those who had uncontrolled BP, which may lead to an improvement in antihypertensive drug treatment [56]. On the other hand, people with hypertension in the intervention group had significantly improved antihypertensive drug-titration rate at T1 and T2 (*p*=0.01 and <0.001, respectively). Although usual care (with or without PFPI) revealed effect on improving antihypertensive drug-treatment rate, the PFPI together with usual care for the intervention group had a much better effect on the antihypertensive drug-titration rate.

Evaluating the efficacy of antihypertensive drugs in BP control (e.g., BP monitoring) promptly is a prerequisite for drug titration [34]. However, only 24.5% to 34.3% of people with hypertension living in China rural communities conducted weekly BP measurements [57]. People with hypertension receiving the PFPI had more frequent BP monitoring (e.g., measured BP at each session) than those in the usual care group, which may lead to a higher antihypertensive drug-titration rate. A recent systematic review examined the effects of family involvement in hypertension care on managing medications of older patients with chronic illnesses [56]. The findings indicated that family carers' participation in conveying information about patients' medication adherence and BP values to healthcare providers and receiving feedback and decision-making in managing medications could lead to drug titration more effectively. The intervention combining BP self-monitoring and self-titration to adjust antihypertensive medication could lead to the reduction of 9.2 mmHg in SBP and 3.4 mmHg in DBP for people with hypertension compared with the usual care group [58]. In the current study, we did not collect the data on dose and types of antihypertensive drugs, and the BP values changed after the drug titration. Therefore, the effect of drug titration on BP change remains unknown. Further studies should collect the data of doses and types of antihypertensive drugs used and changed over time, and the BP values changed after the drug titration, to identify the trajectory of BP change.

#### 4.1.5. Depression and Anxiety Symptoms

The findings of this study revealed that PFPI reduced depressive symptoms, but the effect was nonsignificant between groups. However, anxiety symptoms were significantly reduced at T1 and T2 compared with the control group, with medium and small effect sizes (Cohen's *d* = 0.52 and 0.48, respectively). Inconsistent with the findings on depressive symptoms of hypertensive people in the current study, systematic reviews in chronic illness care have provided evidence of the positive effects of family involvement in illness management programs for adults with different chronic illnesses (e.g., stroke survivors and patients with cancer) [59, 60]. The inconsistency may be due to the low level of people's depression at baseline (mean PHQ-9 score was 4.00), where a PHQ-9 score below 10 is considered to be mild in depression symptoms. Such flooring effect of low depressive symptoms at baseline might cause difficulty in showing treatment effects by making statistically significant changes or improvements in their scores. Patients with serious illnesses (e.g., stroke and cancer) often experience more depressive symptoms [47]. The effect of PFPI on reducing hypertensive people' s depressive symptoms should be further studied.

Increasing evidence of the association between anxiety and hypertension exists [61, 62], and anxiety could negatively affect medication adherence [63]. A significant correlation between anxiety, family function, and QoL was found, and anxiety had a partial mediating effect on the relationship between family function and QoL of older adults with hypertension in low-income communities [64]. The effects of family involvement in hypertension care on anxiety could not be found in previous studies. In other chronic illnesses, such as heart diseases, the effect of family dyad intervention on anxiety in patients with coronary heart disease and family partners was evaluated in a systematic review; the authors could not find significantly lower anxiety levels for patients who received interventions focusing on disease counselling and improving social support than the control group [65]. By contrast, another systematic review demonstrated that interventions targeting the relationship between patients with cancer and their family carers could significantly improve anxiety, depression, and/or distress in both [59]. The potentially effective components of these included studies were education and training about disease knowledge, problem-solving skills, and counselling and discussions in a dyadic manner, similar to the PFPI in the current study. Therefore, even though the evidence of family-dyadic partnership interventions on anxiety symptoms of hypertension and other chronic illnesses (e.g., hypertension, diabetes, and stroke) was limited, the findings of this study supported that family-dyadic partnership intervention could be a promising intervention in improving the anxiety symptoms of people with chronic illness.

#### 4.1.6. Health-Related Quality of Life, HRQoL

PFPI significantly improved the HRQoL of people with hypertension at T1 and T2, with medium and small effects (measured by EQ-5D-5L index: Cohen's *d* = 0.66 and 0.49, respectively; measured by EQ-VAS: Cohen's *d* = 0.61 and 0.51, respectively). An effective family-dyadic partnership was beneficial for QoL of people with hypertension in low-income communities [64]. The structure of SCM also supports a positive relationship between self-care, dyadic relationship, and HRQoL [24]. Similarly, Reid et al. reviewed [63] psychological interventions for patients with coronary heart disease, and their partners demonstrated improvements in the HRQoL of patients at 6 to 13 months postintervention. The main components of the interventions in the reviewed studies were similar to those in this study, including information giving and counselling in 2 to 8 dyadic sessions within 1.5 to 4 months.

### 4.2. Effects on the Outcomes of Family Carers

#### 4.2.1. Dyadic Relationship Quality

With no available evidence for the family's perceived dyadic relationship in hypertension care, the GEE test identified statistically significant interaction effects of PFPI on the family carers' perceived dyadic relationship at T1 (*p*=0.04) and T2 (*p*=0.049) with small effect sizes (Cohen's *d* = 0.36 and 0.31, respectively). In two pilot studies (with small sample sizes of four and 10 only), shared care interventions developed based on SCM were tested among patients with heart failure and their family carers. These studies reported improvements in the quality of dyadic relationship (also measured by DRS-CG) immediately post-intervention [27, 28]. Dyadic communication and reciprocity in healthcare were the main contents of PFPI in the current study, leading to significant improvements in dyadic relationship. Family-dyadic partnership-focused intervention had positive effects on improving the dyadic relationships in their verbal feedback, such as knowing each other better, expressing suggestions and assistance positively, exchanging views on illness experiences, and clarifying perceptions [50]. The findings could support further research to employ the patient-family (carer) partnership model of intervention to improve the dyadic relationship of family carers and patients with hypertension.

#### 4.2.2. Depressive and Anxiety Symptoms

The PFPI has positively improved the family carers' depressive symptoms, but the GEE test results did not indicate statistically significant effect between groups. None of the studies reviewed in our previous systematic review provided evidence about the family carers' depressive symptoms in hypertension care. The result of a systematic review and meta-analysis demonstrated that a stronger perception of filial obligation was associated with increased depressive symptoms among family carers, especially in Chinese culture [66]. Family dyadic relationship-focused interventions can significantly improve family carers' depression of patients with stroke, cancer, and arthritis [59]. Since patient and family carer depression influences patient self-care and family carer's contribution to self-care, healthcare providers should assess and treat depression in both members of the dyad to improve self-care [67]. However, caring for seriously ill patients (e.g., stroke and cancer) can bring more caregiving burden and pressure to family care. As discussed above, the low level of family carers' depression at baseline might cause difficulty in showing treatment effects by making statistically significant changes or improvements.

In the current study, family carers' anxiety symptoms were significantly improved at T2, with a small effect size (Cohen's *d* = 0.28). The improved dyadic partnership is conducive to lowering anxiety symptoms, as declared in SCM [24]. No information on anxiety symptoms of family carers for people with hypertension could be obtained from previous studies. The positive effect of interventions involving the family in treatment of the anxiety of family carers has been demonstrated in systematic reviews of patients with other chronic physical diseases, such as cardiovascular disease, arthritis, and diabetes [68]. An “umbrella” review also found that dyadic relationship-focused family interventions could improve the mental health of adult patients with chronic illness and their family carers [47]. Therefore, family-dyadic partnership/relationship intervention may positively affect the family carers' anxiety symptoms, and further research is needed to support the positive effect of PFPI in improving the anxiety symptoms of family carers in hypertension care.

#### 4.2.3. Health-Related Quality of Life (HRQoL)

PFPI significantly improved the HRQoL of family carers at T1 and T2 (*p*=0.03 and 0.02, respectively), with small effects when measured using EQ-5D-5L index (both Cohen's *d* = 0.44). However, the difference in HRQoL measured using EQ-VAS was statistically significant at T1 only (*p*=0.04) with a small effect size (Cohen's *d* = 0.33). The symptoms, mood, knowledge, and behaviour of people with hypertension towards hypertension management and the family carers' caregiving burden were significantly related to the HRQoL of the family carer [69]. The results of a systematic review and meta-analysis that showed lower poststroke cognitive performance were associated with poorer caregivers' quality of life [68]. In the current study, with the positive effects of PFPI in improving patients' self-care, anxiety symptoms, and family carers' perceived dyadic relationship, the improvement of HRQoL could also be statistically significant and positive. Similar to other family outcomes used in this study, limited evidence was available in previous hypertension care studies. Further research is suggested to confirm the effect of PFPI on improving the carer's HRQoL.

### 4.3. Limitations

Several limitations should be acknowledged. First, the participants in this study were recruited in only two villages in Hunan Province in Southern China, thus limiting the representativeness of the participants and the generalisability of this study to other rural areas in China. Second, this study examined the short-term (one- and three-month postintervention) effects of PFPI on the outcome variables of people with hypertension and their family carers. The longer-term (e.g., six months or above) effects of PFPI on these outcomes remain unknown, and they need to be tested in further studies. Third, although a checklist of all items of the intervention protocol was used to monitor the intervention fidelity by the researcher during each session, this study lacked sufficient process evaluation. Future studies are recommended to adopt a process evaluation to thoroughly understand the intervention implementation, compliance of participants, and successful features of the programme [70]. An independent observer or audio and video recording could be used to measure implementation fidelity. Lastly, as an individual's BP values could fluctuate throughout the day, the BP values obtained from measuring at a one time point on the data collection day may not comprehensively reflect the patient's BP controls. A 24-hour ambulatory blood pressure monitoring could be adopted in future research to obtain more accurate BP values.

### 4.4. Implications

This study provides evidence on integrating the PFPI into hypertension management programmes for rural communities in China. This study also reveals its potential application to a wider hypertensive population in the rural and urban areas of China. Several suggestions were provided to facilitate the implementation of PFPI in practice. First, the study findings could help health policymakers and government administrators realise the importance of patient-family (carer) partnership as the model of intervention in hypertension management in communities, particularly in rural areas with very limited healthcare service. They could also promote the related health regulations or policies to emphasise the adoption of patient-family partnership models of care in community-based hypertension management or services. Second, hypertension care stakeholders need to discuss the barriers and facilitators of integrating PFPI into the current hypertension management service. Third, the village doctors and/or social workers, who are the common healthcare workers in rural areas, should be trained to conduct PFPI.

## 5. Conclusion

We evaluated the effectiveness of PFPI on people with hypertension and their family carers in rural communities in China. The findings demonstrated statistically significant positive effects of PFPI on the majority of the outcomes of participants. These findings supported integrating PFPI into the current hypertension care programmes/services as it could be a more feasible and effective approach to hypertension management in rural areas of China. Further studies are recommended to apply a mixed-method research design and recruit participants with diverse clinical, sociodemographic, and ethnic backgrounds to evaluate the longer-term effects of PFPI in different China regions.

## Figures and Tables

**Table 1:** Protocol of the patient-family carer partnership intervention (PFPI).

Session no. and name	Week no.	Time (min)	Main contents
Session 1 information giving	Week 1	30	(i) Measure BP
			(ii) Inform dyad about the pharmacological therapy
			(iii) Inform dyad about criteria of healthy behaviours regarding hypertension management
			(iv) Guide the dyad to identify the patient's problem behaviours
			*Tool*: Hypertension management booklet (see supplementary file 2)

Session 2 communication skills training	Week 3	30	(i) Measure BP
			(ii) Dyad reports the progress of behaviour modification
			(iii) Discuss with the family carer about issues in caring for and living with a family member with hypertension and his/her role as a family carer in hypertension management
			(iv) Communication skills training
			(1) Encourage the patient to share his/her symptoms and feelings
			(2) Use didactic teaching and role-playing with case scenario to train dyad's communication skills in behaviour change, including listening fully and actively, reducing controlling, criticizing, or guilt provoking language, providing the rationale for self-management behaviours, expressing empathy and concern, enhancing choice, and expressing appreciation
			(v) Encourage the family carer (using the learned communication skills) to help patient implement behaviour change in daily life
			*Tool*: Case scenarios

Session 3 decision-making techniques training	Week 5	30	(i) Measure BP
			(ii) Dyad reports the progress of behaviour modification
			(iii) Assess dyad's implementation of learned communication techniques in daily hypertension care and session
			(1) Ask the family carer to provide examples of using the learned communication techniques in hypertension care
			(2) Observe the dyad's communication in session to confirm their communication issues
			(3) Discuss the issues of using communication techniques in hypertension care in the recent two weeks
			(4) Further training of communication techniques is provided with additional case scenarios and by further discussion
			(5) The identified communication issues are recorded in the “checklist of partnership skills implementation in sessions and daily life”
			(iv) Decision-making techniques training
			(1) Identify problem behaviours and set goals
			(2) Discuss the influence of behaviour change
			(3) Discuss the difficulties of behaviour change and identify the coping strategy
			(4) Discuss ways to seek information and help when needed
			(5) Identify an action plan. The dyad is guided by an “action plan for behaviour change worksheet” to develop the plan for actions
			(v) Encourage the dyad to implement the decision-making techniques and the action plan in daily life
			*Tools*: An action plan for behaviour change worksheet; checklist of partnership skills implementation in sessions and daily life

Session 4 reciprocity techniques training	Week 7	30	(i) Measure BP
			(ii) Dyad reports the progress of behaviour modification
			(iii) Reciprocity techniques training
			(1) Discuss the existing and potential assistance provided by the family carer and received by the patient in behaviour change
			(2) Discuss issues of giving and receiving assistance and explore the strategies
			(3) Express gratitude. Dyad is asked to present his/her obtained achievements and to express their congratulations and gratitude to each other for their achievements
			(iv) Assess dyad's implementation of learned decision-making techniques in hypertension care
			(v) Encourage dyad to implement reciprocity techniques in hypertension care
			*Tools*: An action plan for behaviour change worksheet; checklist of partnership skills implementation in sessions and daily life

Session 5 review	Week 9-10	30	(i) Measure BP
			(ii) Dyad reports the progress of behaviour modification
			(iii) Assess the implementation of learned reciprocity techniques in daily life
			(iv) Review
			(1) Review the contents of the last four sessions
			(2) Review the issues of building a positive partnership that arose in previous sessions
			*Tools*: Hypertension management booklet; an action plan for behaviour change worksheet; checklist of partnership skills implementation in sessions and daily life

**Table 2:** Baseline characteristics of people with hypertension in the intervention and control groups (*n* = 110).

Characteristics	Intervention group (*n* = 55)	Control group (*n* = 55)	*χ* ^2^/*t*	*p*
Gender			*χ* ^2^ = 0.34	0.56
Male	23 (41.82)	20 (36.36)		
Female	32 (58.18)	35 (63.64)		
Age (year)	67.24 ± 12.33	68.05 ± 10.75	*t* = 0.37	0.71
Marital status			*χ* ^2^ = 0.73	0.39
Married	37 (67.27)	42 (76.36)		
Single/Divorced/Widowed	17 (32.73)	13 (23.64)		
Job nature				1.00^†^
Farmer	53 (96.36)	53 (96.36)		
Others (e.g., businessman or factory worker)	2 (3.64)	2 (3.64)		
Educational level			*χ* ^2^ = 1.09	0.58
Illiteracy	17 (30.91)	16 (29.09)		
Primary school	31 (56.36)	35 (63.64)		
Secondary or above	7 (12.73)	4 (7.27)		
Body mass index (BMI)^@^	23.43 ± 3.52	23.61 ± 3.73	*t* = 0.27	0.79
Alcohol drinking			*χ* ^2^ = 0.16	0.92
Never	35 (63.64)	37 (67.27)		
Quitted (≧6 months)	11 (20)	10 (18.18)		
Currently drinking	9 (16.36)	8 (14.55)		
Smoking			*χ* ^2^ = 1.03	0.60
Never	30 (54.55)	35 (63.64)		
Quitted (≧6 months)	3 (5.45)	3 (5.45)		
Currently smoking	22 (40)	17 (30.91)		
Did you do farm work at least once in the last seven days?			*χ* ^2^ = 0	1.00
Yes	30 (54.55)	30 (54.55)		
No	25 (45.45)	25 (45.45)		
Did you have physical exercises at least once in the last seven days?				1.00^†^
Yes (running/jogging)	2 (3.64)	1 (1.82)		
No	53 (96.36)	54 (98.18)		
Annual family income (Yuan)^§^			*χ* ^2^ = 5.52	0.06
10,000–20,000^^^	18 (32.73)	12 (21.82)		
20,000–50,000	25 (45.45)	37 (67.27)		
≥50,000	12 (21.82)	6 (10.91)		
Family structure			*χ* ^2^ = 0.15	0.70
Nuclear family^*∗*^	22 (40)	24 (43.64)		
Stem family^#^	33 (60)	31 (56.36)		
Medical insurance				
New cooperative medical scheme^&^	55 (100%)	55 (100%)		
Duration of hypertension (year)	6.58 ± 3.73	5.91 ± 3.55	*t* = - 0.97	0.34
Number of comorbidities			*χ* ^2^ = 0.63	0.73
0	20 (36.36)	23 (41.82)		
1	29 (52.73)	28 (51.91)		
≥2	6 (10.91)	4 (7.27)		
Diabetes mellitus			*χ* ^2^ = 0.71	0.40
Yes	18 (32.73)	14 (25.45)		
No	37 (67.27)	41 (74.55)		
Have you taken antihypertensive drugs in the past two months?			*χ* ^2^ = 1.33	0.25
Yes	34 (61.82)	28 (50.91)		
No	21 (38.18)	27 (49.09)		

Note. ^@^BMI is calculated by dividing the weight in kilograms by the square of the height in meters (kg/m^2^). ^†^Tested by Fisher's exact test. ^§^1 Yuan = 0.15 US dollars. ^^^Two patients with family incomes below 10,000 yuans were included in the “10,000–20,000 yuan” group. ^*∗*^Nuclear family refers to a family group consisting of a married couple with or without children. ^#^Stem family refers to those families being made up of three generations, that is, a couple and a married child with children. ^&^New Cooperative Medical Scheme is a voluntary and governmentally organized scheme largely financed through government subsidisation. It aims to ensure China rural residents can receive basic healthcare services.

**Table 3:** Baseline characteristics of family carers in two study groups (*n* = 110).

Characteristics	Intervention group (*n* = 55)	Control group (*n* = 55)	*χ* ^2^/*t*	*p*
Relationship with the patients			*χ* ^2^ = 1.02	0.31
Spouse	34 (61.82)	39 (70.91)		
Son/son-in-law/daughter/daughter-in-law	21 (38.18)	16 (29.09)		
Gender			*χ* ^2^ = 0.62	0.43
Male	32 (58.18)	36 (65.45)		
Female	23 (41.82)	19 (34.55)		
Age	59.27 ± 9.07	59.75 ± 10.05	*t* = 0.26	0.80
Marital status			*χ* ^2^ = 0.21	0.65^†^
Married	53 (96.36)	52 (94.55)		
Single/Divorced	2 (3.64)	3 (5.45)		
Employment nature			*χ* ^2^ = 1.50	0.22
Farmer	47 (85.45)	51 (92.73)		
Others (e.g., businessman or service workers or workers in factories)	8 (14.55)	4 (7.27)		
Educational level			*χ* ^2^ = 2.33	0.31
Illiteracy	7 (12.73)	4 (7.27)		
Primary school	38 (69.09)	35 (63.64)		
Secondary or above	10 (18.18)	16 (29.09)		
Body mass index (BMI)	24.72 ± 2.83	25.06 ± 2.75	*t* = 0.65	0.52
Alcohol drinking status			*χ* ^2^ = 0.59	0.74
Never	27 (49.09)	23 (41.82)		
Quitted (≧6 months)	12 (21.82)	14 (25.45)		
Currently drinking	16 (29.09)	18 (32.73)		
Smoking status			*χ* ^2^ = 2.44	0.26^†^
Never	23 (41.82)	20 (36.36)		
Quitted (≧6 months)	4 (7.27)	1 (1.82)		
Currently smoking	28 (50.91)	34 (61.82)		
Number of comorbidities			*χ* ^2^ = 0.70	0.74^†^
0		30 (54.55)		
1-2	26 (47.27)	21 (38.18)		
>2	25 (45.45)	4 (7.27)		
Diabetes mellitus			*χ* ^2^ = 0.53	0.47
Yes	4 (7.27)	12 (21.82)		
No	9 (16.36) 46 (83.64)	43 (78.18)		

Note. ^†^Tested by Fisher's exact test.

**Table 4:** Outcome variables of the hypertensive patients and family carers in PFPI and control groups at T0, T1, and T2.

Outcomes (instruments)	PFPI (mean ± SD)	Control group (mean ± SD)	Effect size (Cohen's *d*/OR)	Comparison of groups at T0	
				*t*/*χ*^2^	*p*
Patients:					
SBP					
T0	154.51 ± 9.43	156.6 ± 10.99		1.07	0.29
T1	144.56 ± 9.48	153.50 ± 13.38	0.77 (medium)		
T2	141.44 ± 8.90	151.18 ± 12.02	0.92 (large)		
DBP					
T0	82.27 ± 10.47	83.78 ± 12.67		0.68	0.50
T1	77.91 ± 8.68	82.39 ± 11.63	0.44 (small)		
T2	76.96 ± 8.41	81.75 ± 11.74	0.47 (small)		
People with normal BP					
T0	0	0			
T1	13 (23.64%)	9 (16.36%)	1.58		
T2	23 (41.82%)	9 (16.36%)	3.67		
Dyadic-relationship quality (DRS-C-PT)					
T0	12.60 ± 3.45	12.22 ± 3.90		−0.55	0.58
T1	10.48 ± 2.75	12.22 ± 3.89	0.52 (medium)		
T2	10.06 ± 2.73	12.16 ± 3.70	0.65 (medium)		
Self-care (HBP SCP-Behaviour)					
T0	45.87 ± 5.35	46.89 ± 5.88		0.95	0.34
T1	52.26 ± 5.84	48.04 ± 6.35	0.69 (medium)		
T2	52.06 ± 5.68	48.24 ± 5.99	0.65 (medium)		
Self-efficacy (HBP SCP-Self-efficacy)					
T0	52.84 ± 5.75	53.35 ± 6.64		0.43	0.67
T1	56.06 ± 5.85	53.94 ± 7.08	0.33 (small)		
T2	56.81 ± 6.28	54.65 ± 7.46	0.31 (small)		
Antihypertensive drug-treatment rate^#^					
T0	29 (52.73%)	31 (56.36%)		0.15	0.70
T1	38 (69.09%)	33 (60.00%)	1.49		
T2	39 (70.91%)	34 (61.82%)	1.51		
Hypertensive drug-titration rate^#^					
T0	7 (12.73%)	5 (9.09%)		0.38	0.54
T1	17 (30.91%)	2 (3.64%)	11.81		
T2	20 (36.36%)	3 (5.45%)	9.91		
Depressive symptoms (PHQ-9)^*∗*^					
T0	4.00 (3.00–6.00)	4.00 (3.00–7.00)		0.73	0.47
T1	3.00 (2.00–5.00)	3.50 (2.00–6.25)	0.48 (small)		
T2	3.00 (2.00–5.00)	3.00 (2.00–6.00)	0.33 (small)		
Anxiety symptoms (GAD-7)^*∗*^					
T0	2.00 (2.00–4.00)	3.00 (2.00–4.00)		0.80	0.43
T1	2.00 (1.00–3.00)	2.00 (2.00–4.00)	0.52 (medium)		
T2	2.00 (1.00–2.75)	2.00 (2.00–4.00)	0.48 (small)		
Health-related quality of life (EQ-5d-5L index score)					
T0	0.88 ± 0.06	0.88 ± 0.08		−0.46	0.65
T1	0.92 ± 0.05	0.88 ± 0.07	0.66 (medium)		
T2	0.92 ± 0.05	0.89 ± 0.07	0.49 (small)		
Health-related quality of life (EQ-VAS)					
T0	72.16 ± 8.91	70.18 ± 10.41		−1.07	0.29
T1	76.35 ± 7.78	71.30 ± 8.67	0.61 (medium)		
T2	76.50 ± 7.97	72.16 ± 8.89	0.51 (medium)		
Family carers:					
Dyadic-relationship quality (DRS-C-CG)					
T0	15.02 ± 5.35	14.76 ± 4.48		−0.27	0.79
T1	12.50 ± 4.24	14.06 ± 4.36	0.36 (small)		
T2	12.15 ± 4.00	13.47 ± 4.63	0.31 (small)		
Depressive symptoms (PHQ-9)					
T0	2.65 ± 1.42	2.69 ± 1.57		0.13	0.90
T1	2.13 ± 1.21	2.37 ± 1.39	0.18 (small)		
T2	2.04 ± 1.10	2.41 ± 1.42	0.29 (small)		
Anxiety symptoms (GAD-7)					
T0	2.27 ± 1.31	2.09 ± 1.67		−0.64	0.53
T1	1.69 ± 0.97	2.17 ± 1.22	0.44 (small)		
T2	1.54 ± 0.96	1.86 ± 1.30	0.28 (small)		
Health-related quality of life (EQ-5D-5L index score)					
T0	0.92 ± 0.06	0.92 ± 0.06		0.94	0.95
T1	0.94 ± 0.04	0.92 ± 0.05	0.44 (small)		
T2	0.94 ± 0.04	0.92 ± 0.05	0.44 (small)		
Health-related quality of life (EQ-VAS)					
T0	75.45 ± 8.73	75.42 ± 9.39		−0.02	0.98
T1	78.70 ± 7.66	76.13 ± 7.82	0.33 (small)		
T2	78.83 ± 7.61	77.33 ± 7.60	0.20 (small)		

Note. OR, odds ratio; DRS-C-PT, Chinese version of Dyadic Relationship Scale (patient version); DRS-C-PT, Chinese version of Dyadic Relationship Scale (caregiver version); HBP SCP-Behaviour, Behaviour scale of Hypertension Self-Care Profile; HBP SCP-self-efficacy, Self-efficacy of Hypertension Self-Care Profile; PHQ-9, Patient Health Questionnaire-9; GAD-7, Generalised Anxiety Disorder Scale-7; EQ-5D-5L, EuroQol five-dimensional five-level; EQ-VAS, EuroQol Visual Analogue scale. ^#^Presented as *n* (%). ^*∗*^Presented as median (lower quartile, upper quartile).

**Table 5:** Generalised estimating models for comparing the outcomes across time between the intervention and control groups.

Outcomes	Group effect		Time effect		Group × time effect			Pairwise contrast test			
	*β* (95% CI)	*p*	*β* (95% CI)	*p*	*β* (95% CI)	Wald *χ*^2^	*p*	T1		T2	
								MD(95% CI)	*p*	MD(95% CI)	*p*
*People with hypertension*											
SBP	−2.06 (−4.78, 0.66)	<0.001^*∗∗∗*^	−4.68 (−6.90, −2.46)	<0.001^*∗∗∗*^	−8.01 (−11.30, −4.71)	25.98	<0.001^*∗∗∗*^	−8.92 (−13.22, −4.62)	<0.001^*∗∗∗*^	−10.10 (−14.11, −6.09)	<0.001^*∗∗∗*^
DBP	−1.51 (−5.81, 2.79)	0.07	−2.08 (−3.52,−0.65)	<0.001^*∗∗∗*^	−3.15 (−5.12, −1.18)	20.52	<0.001^*∗∗∗*^	−4.84 (−8.65, −1.03)	0.01^∗^	−4.66 (−8.60, −0.72)	0.02^∗^
Proportion of people with normal BP	1.59 (0.61, 4.10)	0.04^∗^	1.02 (0.59, 1.76)	0.02^∗^	2.38 (1.09, 5.20)	4.70	0.03^∗^	0.07(−0.08, 0.23)	0.36	0.27 (0.08, 0.46)	0.01^∗^
DRS-C-PT	0.44 (−0.81, 1.68)	0.04^∗^	0.13 (−0.24, 0.49)	<0.001^*∗∗∗*^	−2.77 (−3.33, −2.21)	109.30	<0.001^*∗∗∗*^	−1.72 (−2.89, −0.55)	0.004^*∗∗*^	−2.34 (−3.50, −1.17)	<0.001^*∗∗∗*^
HBP SCP- behaviour	−0.02 (−2.99, 0.95)	0.02^∗^	1.30 (0.60, 2.00)	<0.001^*∗∗∗*^	4.91 (3.94, 5.87)	138.07	<0.001^*∗∗∗*^	4.30 (2.13, 6.46)	<0.001^*∗∗∗*^	3.89 (1.82, 5.95)	<0.001^*∗∗∗*^
HBP SCP- self-efficacy	−0.51 (−2.70, 1.79)	0.20	0.88 (0.01, 1.75)	<0.001^*∗∗∗*^	3.24 (2.01, 4.47)	29.78	<0.001^*∗∗∗*^	2.21 (−0.12, 4.54)	0.06	2.73 (0.24, 5.22)	0.03^∗^
Antihypertensive drug-treatment rate	0.95 (0.45, 1.99)	0.34	1.42 (1.04, 1.92)	<0.001^*∗∗*^	1.90 (1.02, 3.53)	5.50	0.06	0.13 (−0.05, 0.31)	0.17	0.12 (−0.05, 0.29)	0.18
Hypertensive drug-titration rate	1.43 (0.42, 4.87)	<0.001^*∗∗∗*^	0.58 (0.12, 2.69)	0.53	7.00 (1.11, 44.22)	4.96	0.08	0.27 (0.08, 0.46)	0.01^∗^	0.31 (0.12, 0.49)	0.001^*∗∗*^
PHQ-9	−0.01 (−0.08, 0.06)	0.25	−0.01 (−0.04, 0.01)	<0.001^*∗∗∗*^	−0.04 (−0.08, −0.01)	6.70	0.04^∗^	−0.04 (−0.10, 0.02)	0.21	−0.06 (−0.12, 0.01)	0.08
GAD-7	−0.03 (−0.09, 0.03)	0.03^∗^	−0.02 (−0.04,−0.01)	<0.001^*∗∗∗*^	−0.04 (−0.08, −0.01)	6.84	0.03^∗^	−0.07 (−0.11, −0.02)	0.01^∗^	−0.07 (−0.12, −0.02)	0.01^∗^
EQ-5D-5L index score	0.01 (−0.02, 0.03)	0.05	0.01 (0.00, 0.02)	<0.001^*∗∗∗*^	0.02 (0.00, 0.04)	18.76	<0.001^*∗∗∗*^	0.03 (0.01, 0.06)	0.003^∗^	0.03 (0.01, 0.05)	0.01^∗^
EQ-VAS	1.98 (−1.32, 5.28)	0.01^*∗∗*^	1.34 (0.19, 2.48)	<0.001^*∗∗∗*^	3.03 (1.45, 4.61)	20.89	<0.001^*∗∗∗*^	5.20 (2.34, 8.06)	<0.001^*∗∗∗*^	5.02 (2.00, 8.03)	0.001^*∗∗*^

*Family carers*											
GRS-C-CG	0.26 (−1.49, 2.00)	0.23	−1.14 (−1.59,−0.68)	<0.001^*∗∗∗*^	−1.84 (−2.58, −1.10)	34.16	<0.001^*∗∗∗*^	−1.61 (−3.17, −0.06)	0.04^∗^	−1.59 (−3.17, −0.01)	0.049^∗^
PHQ-9	−0.04 (−0.58, 0.50)	0.31	−0.26 (−0.56, 0.04)	<0.001^*∗∗∗*^	−0.37 (−0.72, −0.01)	4.10	0.13	−0.27 (−0.75, 0.20)	0.26	−0.40 (−0.87, 0.06)	0.09
GAD-7	0.18 (−0.37, 0.74)	0.37	−0.23 (−0.48, 0.02)	<0.001^*∗∗∗*^	−0.47 (−0.80, −0.14)	15.69	<0.001^*∗∗∗*^	−0.48 (−0.88, −0.07)	0.02^∗^	−0.29 (−0.72, 0.14)	0.19
EQ-5D-5L index score	0.00 (−0.02, 0.02)	0.11	−0.00 (−0.01, 0.01)	0.05	0.02 (0.01, 0.04)	7.25	0.03^∗^	0.02 (0.00, 004)	0.03^∗^	0.02 (0.00, 004)	0.02^∗^
EQ-VAS	0.04 (−3.14, 3.21)	0.23	1.55 (0.44, 2.66)	0.001^*∗∗*^	2.11 (0.48, 3.73)	15.22	<0.001^*∗∗∗*^	2.90 (0.18, 5.63)	0.04^∗^	2.14 (−0.59, 4.87)	0.12

Note. DRS-C-PT, Chinese version of Dyadic Relationship Scale (patient version); DRS-C-PT, Chinese version of Dyadic Relationship Scale (caregiver version); HBP SCP-Behaviour, Behaviour scale of Hypertension Self-Care Profile; HBP SCP-self-efficacy, Self-efficacy of Hypertension Self-Care Profile; PHQ-9, Patient Health Questionnaire-9; GAD-7, Generalised Anxiety Disorder Scale-7; EQ-5D-5L, EuroQol five-dimensional-five-level; EQ-VAS, EuroQol Visual Analogue scale. ^∗^*p* < 0.05; ^∗∗^*p* < 0.01; ^∗∗∗^*p* < 0.001.

## Data Availability

The datasets used to support the findings of this study are available from the corresponding author upon request.

## Additional Points

Reporting Method. The study is reported in line with the Consolidated Standards of Reporting Trials 2010 Statement. Patient or Public Contribution. Eligible hypertensive people receiving home visits and care at two village clinics and their family carers were randomly enrolled in this study. The researcher reviewed the hypertensive people's medical records in the two village clinics under study and created a list of potential participants (those aged 18 years or above and having essential hypertension without adequate BP control) in alphabetical order of their family names after screening. The people on the list were randomly selected using a random number table and approached by the researcher during home visits to further confirm the study eligibility (living with one or more adult family members, speaking Mandarin or local dialect, and without terminal and mental illness, etc.). The researcher introduced the study to the participants and the benefits, risks, participants' rights, and confidentiality when participating in this study. Written informed consent was obtained from the participants who agreed to participate in this study. The researcher delivered the intervention to participants in the intervention group. A retired village doctor was trained to collect data. What Does this Paper Contribute to the Wider Global Clinical Community? (i) The patient-family (carer) partnership intervention has the potential to improve hypertensive people's BP control in rural areas. (ii) The intervention also helps to improve hypertensive people's and family carer's dyadic-relationship quality and mental health at short-to-medium-term follow-ups. (iii) This study provides evidence on integrating the patient-family (carer) partnership intervention into hypertension management programmes for rural communities worldwide. Trial Registration. This study was registered in the Chinese Clinical Trial Registry https://www.chictr.org.cn (registration number: ChiCTR1900027087) in October 2019, and the first participant was recruited in July 2020.

## Supplementary Materials

Supplementary Materials Supplementary material 1: a checklist of intervention fidelity. The checklist consisted of all items of the intervention protocol. It was used by the researcher during each intervention session to monitor the intervention fidelity. At the end of each session, the researcher checked the completion of each item on the list with family dyad and marked as “completed” or “uncompleted.” The “uncompleted” intervention item was made up in time. Supplementary material 2: hypertension management booklet. The booklet comprised four sections, including the goal of BP control, risk assessment for cardiovascular disease, criteria of healthy behaviours and worksheets for identifying behavioural problems, and hypertension self-care.
