# Supplementary material for: Effectiveness of a Patient-Family Carer Partnership Intervention on Blood Pressure Control for People with Hypertension in Rural Communities: A Randomised Controlled Trial
Source: J Nurs Manag. 2024 May 24;2024:7033013. doi: 10.1155/2024/7033013 (PMC11918710; doi:10.1155/2024/7033013)
Supplement: Supplementary Materials — Supplementary material 1: a checklist of intervention fidelity. The checklist consisted of all items of the intervention protocol. It was used by the researcher during each intervention session to monitor the intervention fidelity. At the end of each session, the researcher checked the completion of each item on the list with family dyad and marked as “completed” or “uncompleted.” The “uncompleted” intervention item was made up in time. Supplementary material 2: hypertension management booklet. The booklet comprised four sections, including the goal of BP control, risk assessment for cardiovascular disease, criteria of healthy behaviours and worksheets for identifying behavioural problems, and hypertension self-care. [file 7033013.f1.zip › Supplemental material 1.doc]

A checklist of intervention fidelity

| **Sessions and contents** | **Complete status^a^** | **Remarks** |
| --- | --- | --- |
| **Pre-intervention** |  |  |
| Eligibility assessment |  |  |
| Obtain written consent |  |  |
| Baseline data collection |  |  |
| Randomization |  |  |
| **Session 1** |  |  |
| Measure BP |  |  |
| Information giving |  |  |
| Problem behaviors identification |  |  |
| **Session 2** |  |  |
| Measure BP |  |  |
| Dyad report behavior change progress |  |  |
| Discussion on family carer’s experience and roles in hypertension care |  |  |
| **Communication skills training:** | | |
| - Patient’s symptoms and feeling sharing |  |  |
| - Didactic teaching |  |  |
| - Role-playing |  |  |
| Homework |  |  |
| **Session 3** |  |  |
| Measure BP |  |  |
| Dyad report behavior change progress |  |  |
| Assess dyad’s implementation of learned communication techniques |  |  |
| **Decision-making techniques training:** | | |
| - Identify problem behaviors and set goals |  |  |
| - Discuss the influence of behavior change |  |  |
| - Discuss the difficulties of behavior change and identify the coping strategy |  |  |
| - Discuss ways to seek information and help |  |  |
| - Identify an action plan |  |  |
| Homework |  |  |
| **Session 4** |  |  |
| Measure BP |  |  |
| Dyad report behavior change progress |  |  |
| **Reciprocity techniques training:** | | |
| - Discuss the existing and potential assistance provided and received by dyads in behavior change |  |  |
| - Discuss issues of giving and receiving assistance and explore the strategies |  |  |
| - Express gratitude |  |  |
| Assess dyad’s implementation of the learned decision-making techniques |  |  |
| Homework |  |  |
| **Session 5** |  |  |
| Measure BP |  |  |
| Dyad report behavior change progress |  |  |
| Assess dyad’s implementation of the learned reciprocity techniques |  |  |
| Review the contents of the last four sessions |  |  |
| Review the issues of building a positive partnership that arose in previous sessions |  |  |
| Inform the date of the next home visit for post-intervention data collection |  |  |

^a^ Completed:√; Uncompleted: X
