# Supplementary material for: Effectiveness of a Patient-Family Carer Partnership Intervention on Blood Pressure Control for People with Hypertension in Rural Communities: A Randomised Controlled Trial
Source: J Nurs Manag. 2024 May 24;2024:7033013. doi: 10.1155/2024/7033013 (PMC11918710; doi:10.1155/2024/7033013)
Supplement: Supplementary Materials — Supplementary material 1: a checklist of intervention fidelity. The checklist consisted of all items of the intervention protocol. It was used by the researcher during each intervention session to monitor the intervention fidelity. At the end of each session, the researcher checked the completion of each item on the list with family dyad and marked as “completed” or “uncompleted.” The “uncompleted” intervention item was made up in time. Supplementary material 2: hypertension management booklet. The booklet comprised four sections, including the goal of BP control, risk assessment for cardiovascular disease, criteria of healthy behaviours and worksheets for identifying behavioural problems, and hypertension self-care. [file 7033013.f1.zip › Supplemental material 2 - English version.doc]

Hypertension Management Booklet

——Lifestyle Modification

**Hypertension**

**What is Hypertension?**

- Hypertension is diagnosed when the office/clinic systolic blood pressure (SBP) is 140 mmHg or above and/or the diastolic blood pressure (DBP) is 90 mmHg or above for untreated individual.
- Anyone who has been diagnosed with hypertension and is currently taking anti-hypertensive medication, blood pressure below 140/90mmHg is also considered hypertension.

**Today, you blood pressure is：______/______ mmHg。**

**Whit the normal blood pressure for people with hypertension？**

- In general, people with hypertension should control their blood pressure below 140/90 mmHg;
- People aged over 65 years old with hypertension should control their blood pressure below 150/90 mmHg;
- People with diabetes, chronic kidney disease with proteinuria should control their blood pressure below 130/80 mmHg.

**I should keep the blood pressure below ______/_______ mmHg.**

**What damages does hypertension have to the body？**

[**cerebral hemorrhage**](javascript:;)

[**cerebral infarction**](javascript:;)

[**coronary heart disease**](javascript:;)

**heart failure**

[**impairment of renal function**](javascript:;)

**impaired vision**

**Has someone close to you caused a cerebral hemorrhage or cerebral infarction due to HYPERTENSION？□ YES	□ NO**

**What damages does hypertension have to MY body？**

**Cardiovascular Risk Assessment**

| Blood pressure (mmHg) | Risk factors | Damages in target organs | Preexisting diseases |
| --- | --- | --- | --- |
| - Level 1：140-159/90-99 - Level 2：160-179/100-109 - Level 3：≥ 180/110 | - age：male≥55 years，female ≥ 65 years - smoking - [dyslipidemia](javascript:;) - Family history of early onset cardiovascular disease: parents, siblings, children, diagnosed with hypertension before 55 years old (male) or 65 years old (female) - Obesity or abdominal obesity | - left ventricular hypertrophy - thickening and plaque of carotid intimedia - impaired kidney function - carotid artery pulse wave conduction velocity ≥12m/s | - cerebrovascular disease - heart disease - kidney disease - peripheral vascular diseases - retinopathy - diabetes |

According to the table above, your risk of cardiovascular disease belongs to**:**

- **Low level：**Level 1 blood pressure without any risk factors；
- **Medium level：**Level 2 blood pressure；or level 1 blood pressure with 1-2 risk factors；
- **High level：**Level 3 blood pressure；Level 1 or 2 blood pressure with more than 3 risk factors; hypertension with 1 target organ damage；or hypertension with preexisting disease.

**How to obtain normal blood pressure？**

**Taking antihypertenive**

**drugs**

**Keeping healthy lifestyles**

- Blood pressure self-monitoring

1

2

3

**Good medication adherence**

- According to medical prescriptions
- Never discontinuing drugs and changing dosages and types without authorisation
- If the blood pressure is not norma after taking antihypertensive drugs for 1 to 3 months, you need to see a doctor to adjust the medication

1

2

3

**Healthy lifestyles**

- sodium restriction, potassium-rich diet, adequate vegetables and fruits, heart-healthy diet
- alcohol restriction, smoking cessation
- regular physical activity, weight control

**What should I do in my daily life？**

1. For sodium restriction, you should：（completed“×”，uncompleted“√”）

□ Limiting daily salt intake below 6 grams (using a beer

cap to roughly estimate the amount)

□Reducing the use of monosodium glutamate ([Ajinomoto](javascript:;)) and soy sauce

□In cooking, more natural seasonings are used, such as scallions, ginger, garlic, spicy pepper, etc., instead of salt.

□ Avoiding or reducing the consumption of foods with high salt content, such as pickles, cured meat, ham and mouldy tofu.

□ Read food labels when buying food and snacks, and choose packaged foods that are low in sodium (salt).

□ Less fast food.

□ Also cut down on salty foods when eating out.

**Eat less, or not at all**

**×**

**×**

**×**

**×**

**×**

（Discussion with family dyad in the second to fifth sessions）

| Barriers in implementing the above recommendations |  |  |  |  |
| --- | --- | --- | --- | --- |
| Measures |  |  |  |  |
| Completion（“√”或“×”）and following strategies |  |  |  |  |

2. For potassium-rich diet, you should：

The recommendation of WHO: daily potassium intake = 3510 mg/day

（except people with chronic renal failure)

□ Potassium is given through diet, not medicine.

□ The consumption of two sets or servings of fruit and three services of vegetables could provide about 3,000 mg potassium.

（Discussion with family dyad in the second to fifth sessions）

| Barriers in implementing the above recommendations |  |  |  |  |
| --- | --- | --- | --- | --- |
| Measures |  |  |  |  |
| Completion（“√”或“×”）and following strategies |  |  |  |  |

3. **A heart-healthy diet for hypertensive people: The Dietary Approaches to Stop Hypertension (DASH)**

| **Food types** | **Amounts/servings** | **Serving** | **Type and amount of food consumed daily for the past 7 days** | **The type amount to be adjusted** |
| --- | --- | --- | --- | --- |
| **Cereals and their products** |  | - One rice bowl cooked rice or other cereals = two servings - One big bowl with soup noodles, rice noodles = two servings - One piece of bread = one serving |  |  |
| **Vegetables** |  | - One rice bowl cooked vegetables = two serving |  |  |
| **Fruits** |  | - One medium size fruit（apple, orange, banana, muskmelon）= one serving - One medium size piece of watermelon = one serving - 3/4 cup of juice = one serving |  |  |
| **Fat-free or low-fat dairy products** |  | - 1.5 cups of milk or yogurt = one serving |  |  |
| **Lean meat, poultry and fish** |  | - Half rice bowl cooked lean meat, poultry or fish = three servings - One egg = one serving |  |  |
| **Nuts and legumes** |  | - Half cup of nuts = one serving - Half a bowl of cooked beans or half a piece of tofu = one serving |  |  |
| **Lipids and edible oils** |  | - One tablespoon vegetable oil (canola, camellia, corn, peanut, etc.)） - Lard is not recommended, and the amount of lard used per day is not more than the thumb size, about 25 grams |  |  |
| **Sugar** |  | - 1.5 flat spoons of white sugar = one serving - 1.5 cups lemonade, Cola, Sprite = one serving |  |  |

- Note: 1 rice bowl = 275 ml; 1 cup = 160 ml; 1 spoon = 5 ml

（Discussion with family dyad in the second to fifth sessions）

| Barriers in implementing the above recommendations |  |  |  |  |
| --- | --- | --- | --- | --- |
| Measures |  |  |  |  |
| Completion（“√”或“×”）and following strategies |  |  |  |  |

4. **For regular physical exercise, your should:**

Moderate-intensity aerobic exercises are recommended:

□ Brisk walking, jogging, physical labour, swimming, cycling, and

square dancing.

□ Four to seven times a week and at least 30 minutes each time.

□ Slow walks and doing housework or household chores were

recommended to some people who were incapable of performing

aerobic exercises.

Discussion with family dyad in the second to fifth sessions）

| Barriers in implementing the above recommendations |  |  |  |  |
| --- | --- | --- | --- | --- |
| Measures |  |  |  |  |
| Completion（“√”或“×”）and following strategies |  |  |  |  |

**5. Alcohol restriction**

□ Drinking is not encouraged.

For those who are currently drinking, the recommended dosage of alcohol intake per day:

□ consume less than 50 ml (i.e., 1 Liang, a unit of capacity measurement commonly used by locals) liquor, 150 ml (3 Liang) red wine, or one beer (350 ml)

□ Female：less than 25 ml (i.e., 0.5 Liang) liquor, 75ml (1.5 Liang) red wine, or half beer (175 ml).

Discussion with family dyad in the second to fifth sessions）

| Barriers in implementing the above recommendations |  |  |  |  |
| --- | --- | --- | --- | --- |
| Measures |  |  |  |  |
| Completion（“√”或“×”）and following strategies |  |  |  |  |

**6. smoking cessation**

□ All hypertensive people were recommended to immediately quit or progressively reduce and stop smoking.

Discussion with family dyad in the second to fifth sessions）

| Barriers in implementing the above recommendations |  |  |  |  |
| --- | --- | --- | --- | --- |
| Measures |  |  |  |  |
| Completion（“√”或“×”）and following strategies |  |  |  |  |

**7.Weight control**.

BMI = weight÷height^2^。

Your BMI is _____ kg/m^2^, then to determine whether it is normal, overweight or obese：

*Normal=18.5～23.9 kg/m^2^；overweight=24.0 ～ 27.9 kg/m^2^；obese ≥ 28 kg/m^2^*

□My BMI is normal.

□My BMI is overweight.

□My BMI is obese.

The DASH approach to diet and regular physical exercise are recommended for weight reduction in overweight or obese patients.

**DASH + Regular physical exercise = weight reduction**

Discussion with family dyad in the second to fifth sessions）

| Barriers in implementing the above recommendations |  |  |  |  |
| --- | --- | --- | --- | --- |
| Measures |  |  |  |  |
| Completion（“√”或“×”）and following strategies |  |  |  |  |

**8.*Medication adherence***

□I don’t need to take antihypertensive drugs according to the medical prescriptions.

□I need to take antihypertensive drugs according to the medical prescriptions, but sometimes I don’t follow it.

Discussion with family dyad in the second to fifth sessions）

| Barriers in implementing the above recommendations |  |  |  |  |
| --- | --- | --- | --- | --- |
| Measures |  |  |  |  |
| Completion（“√”或“×”）and following strategies |  |  |  |  |

**9.BP self-monitoring**

□ An upper-arm electronic sphygmomanometer is recommended for BP monitoring. Anyone who do not have a sphygmomanometer are suggested to visit the village clinic free-of-charge for BP measurement.

□ You should have the skills to measure BP with an electronic BP measurement device at home.。

□ If your BP is abnormal, take one test every morning and one test every night; Continuously measure BP for 7 days.

□ If your BP is normal, take one test every week (one for morning and one for night).
